# Type 3 Diabetes: Linking Insulin Resistance to Cognitive Decline

**DOI:** 10.3390/diseases13110359

**Published:** 2025-11-05

**Authors:** Brooke Chapple, Emily Bayliss, Seth Woodfin, Merritt Smith, Jeremiah Winter, William Moore

**Affiliations:** 1 Department of Biology and Chemistry, School of Health Sciences, Liberty University, Lynchburg, VA 24515, USA; bechapple@liberty.edu (B.C.); egbayliss@liberty.edu (E.B.); mlsmith60@liberty.edu (M.S.); jwinter11@liberty.edu (J.W.); 2 Department of Biomedical Sciences, West Virginia School of Osteopathic Medicine, Lewisburg, WV 24901, USA; swoodfin@osteo.wvsom.edu

**Keywords:** type 3 diabetes, insulin resistance, cognitive decline, Alzheimer’s disease, neurodegeneration, gut–brain axis, oxidative stress, neuroinflammation, synaptic dysfunction, metabolic health

## Abstract

Type 3 diabetes (T3D) is characterized by chronic insulin resistance and insulin deficiency in the brain, leading to neuronal death, inflammation, oxidative stress, apoptosis, and synaptic dysfunction. These pathological processes contribute to cognitive decline and neurodegenerative disorders such as Alzheimer’s disease. However, despite increasing evidence that links insulin resistance to cognitive impairment, the precise mechanisms that underly T3D remain largely unknown. This highlights a critical gap in research and potential therapeutic strategies. Given the significant impact of diet on metabolic health, investigating the correlation between the gut–brain axis may offer novel insights into the prevention and management of T3D. This review aims to elucidate the potential connections between insulin resistance and cognitive decline while also proposing interventions to slow aging and reduce the risk of early cognitive decline. At the same time, we acknowledge that the classification of type 3 diabetes is debatable and there is uncertainty as to whether insulin resistance is a primary driver or secondary manifestation of neurodegeneration.

## 1. Introduction

The concept of type 3 diabetes (T3D) is gaining recognition as a potential link between insulin resistance and cognitive decline, particularly in neurodegenerative diseases such as Alzheimer’s disease (AD), Parkinson’s disease, and dementia. The prevalence of AD doubles every five years among individuals over the age of 65 years, with risk increasing with age [1]. Previously, cognitive decline was considered an inevitable part of aging. However, emerging evidence suggests that insulin resistance plays a critical role in this process, which offers promising avenues for prevention and early intervention. Thus, an understanding of T3D and its relationship to cognitive decline is crucial for developing strategies to preserve cognitive function beyond the age of 65. While the term “type 3 diabetes” has been proposed to describe this overlap, it remains a working hypothesis rather than a universally accepted diagnosis. Some evidence suggests that insulin resistance precedes and exacerbates neurodegeneration, while other data indicate that is could arise secondarily from amyloid and tau pathology [2].

**Figure 1:**
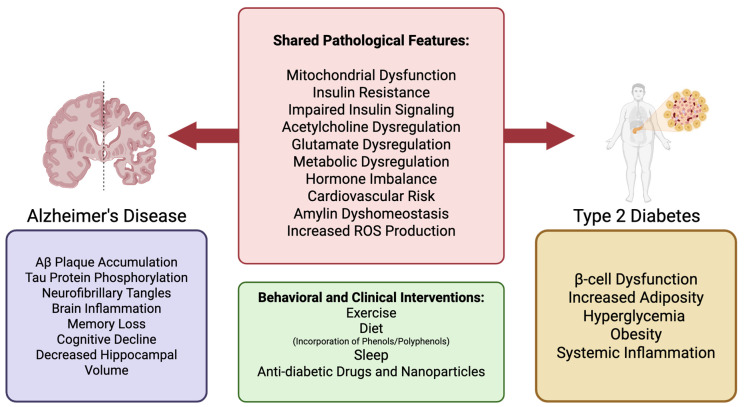
Shared and discrete pathological features of AD and T2D, with potential clinical and behavioral interventions. The left panel illustrates pathological features specific to AD (purple), including Aβ plaque accumulation, tau hyperphosphorylation, and neuroinflammation, which contribute to memory loss and cognitive decline. The right panel highlights T2D-specific features (yellow) such as β-cell dysfunction, obesity, and systemic inflammation. The central panel summarizes overlapping pathologies in both diseases (red), including mitochondrial dysfunction, insulin resistance, glutamate and acetylcholine dysregulation, and increased reactive oxygen species. These shared features suggest a mechanistic link between AD and T2D, supporting the concept of “type 3 diabetes.” The lower box lists behavioral and clinical interventions (green), such as exercise, diet (including phenols/polyphenols), sleep, and anti-diabetic therapies, that may target both diseases through metabolic and inflammatory modulation.

T3D is marked by chronic insulin resistance and insulin deficiency in the brain [3], leading to neuronal cell death, inflammation, oxidative stress, apoptosis, neurodegeneration, synaptic dysfunction, poor dendritic spine formation, and reduced acetylcholine production (Figure 1) [3]. These pathological changes contribute to cognitive decline, memory loss, and impaired executive function. Clinically, this can manifest as depression, aggression, and difficulty performing everyday tasks such as driving, laundry, self-care, and other household activities [4,5]. AD is characterized by the accumulation of beta amyloid (Aβ) plaques and tau protein phosphorylation, leading to neurofibrillary tangles [6]. These factors eventually lead to memory loss, poor problem-solving capabilities, and difficulty completing basic tasks as described above [6]. Impaired insulin signaling is believed to exacerbate these processes, thus emphasizing the connection between insulin resistance and cognitive decline.

Significant gaps remain in our understanding of AD as T3D and its broader implications. The specific mechanisms underlying insulin resistance in the brain and its progression toward T3D are largely unknown, but present promising avenues for both prevention and potential treatment. Several molecular pathways warrant further investigation. For example, insulin regulates γ-aminobutyric acid (GABA)- and glutamate-mediated mechanisms, both of which are essential for long-term potentiation and long-term depression, which are critical processes for learning and memory [7,8]. Insulin also influences the protein kinase B (Akt)/mammalian target of rapamycin (mTOR) and Ras-related pathways, which are crucial for synaptic activity and dendritic spine formation [9].

Neuroinflammation is another significant factor that contributes to cognitive decline and is likely exacerbated by high levels of cytotoxic lipids that contribute to insulin resistance [10]. Chronic positive energy balance and decreased beta oxidation, resulting from overnutrition and mitochondrial dysfunction, respectively, lead to an accumulation of bioactive lipid species such as diacylglycerols and ceramides [11,12,13]. Elevated levels of these cytotoxic lipids cross the blood–brain barrier, triggering neuroinflammation and impairing brain insulin resistance. The inflammatory response leads to increased pro-inflammatory cytokines, such as interleukin-6 (IL-6) and tumor necrosis factor alpha (TNF-α), mirroring the systemic inflammation seen in type 2 diabetes (T2D) [10]. Furthermore, neuroinflammation is associated with the inhibition of the AMP-activated protein kinase (AMPK) pathway, which can be triggered by hyperinsulinemia [14]. These are just a few of the interconnected pathways that highlight the connection between metabolic dysfunction, neurodegeneration, and cognitive decline.

These complex interactions underscore the importance of holistic approaches to health. Diet lifestyle modifications can significantly impact cognition and overall well-being. Small changes, such as increasing antioxidant and omega-3 fatty acid intake while reducing omega-6 fatty acid intake, could potentially slow cognitive decline [15]. Furthermore, the gut–brain axis has emerged as a key area of interest in T3D research, highlighting the potential for dietary interventions to influence brain function. Regular physical activity has also been shown to enhance neurochemical signaling, improve insulin sensitivity, and decrease Aβ plaque accumulation [16]. Similarly, high-quality sleep, especially slow-wave sleep, has been linked to improved hormone regulation, enhanced memory function, and improved insulin signaling, thus offering another avenue for cognitive health interventions.

An improved understanding of the intricate relationship between insulin resistance, T2D, and cognitive decline allows for a more comprehensive approach to the early identification of biomarkers and the development of targeted interventions. By delineating the ripple effects of insulin resistance, especially those related to neurological damage, this work could elucidate potential treatments. Early detection of AD, dementia, and other cognitive disorders through metabolic screening could facilitate the development of optimized and personalized clinical and behavioral interventions, including dietary modifications, supplements, or pharmacological therapies. Viewing the body as an integrated whole, rather than as isolated components, may lead to more effective, root-cause-driven approaches to disease prevention. Strengthening this connection between insulin resistance and cognitive decline can also aid in the design of interventions to slow aging and minimize early cognitive decline, potentially transforming how we approach neurodegenerative diseases in aging populations.

## 2. Shared Pathophysiology of Alzheimer’s Disease and Type 2 Diabetes

### 2.1. Major Hallmarks of Alzheimer’s Disease

The accumulation of Aβ plaques in the brain contributes to dendritic swelling and neuronal cell death [17]. Soluble Aβ oligomers promote inflammation, calcium overload, and neuronal damage, while the Aβ monomers appear to have neuroprotective and antioxidant properties.

The presence of reactive oxygen species (ROS) and Aβ plaques can cause the activation of microglia, causing neuroinflammation and promoting tau phosphorylation [18]. Hyperphosphorylation of tau proteins is likely a key contributor to neurofibrillary tangle formation, which disrupts microtubule stability, ultimately resulting in neuronal death [17]. In addition, tau hyperphosphorylation can impair axonal transport, further exacerbating neuronal dysfunction and degradation [19].

### 2.2. Role of Glutamate and GABA

Glutamate plays a critical role in AD, by inducing excitotoxicity and related oxidative stress [17]. As a primary excitatory neurotransmitter in the brain, glutamate is essential for synaptic plasticity, learning, and memory [20]. Predominantly glutamatergic brain areas include the amygdala, the prefrontal cortex, and the hippocampus [21]. It activates ionotropic glutamate receptors (iGluRs) such as NMDA and AMPA, as well as kainite and metabotropic glutamate receptors (mGluRs). While iGluRs mediate fast synaptic excitation, mGluRs mediate slow excitatory synaptic transmission [22]. These receptors are responsible for indirectly modulation calcium influx in second messenger cascades [23,24]. However, in pathological states, altered function of glutamate receptors can lead to increased neural glutamate sensitivity, promoting the release of excess glutamate [25]. The accumulation of glutamate in the synaptic cleft triggers a massive influx of calcium ions into the neuronal compartment, heightening action potential potentiation [16]. Additionally, elevated intracellular calcium levels further contribute to ROS production, triggering oxidative stress, apoptosis, and a vicious cycle of tau phosphorylation, inflammation, and neuronal death [18]. Furthermore, glutamate plays a role in the regulation of insulin and glucagon secretion and can act as both an intracellular and extracellular signal in pancreatic islet cells. Pancreatic β-cells express NMDA, AMPA, and kainite receptors whereas α-cells express AMPA and kainite receptors [22]. While the exact molecular link between glutamate excitotoxicity and brain insulin resistance remains unclear, glutamate has been demonstrated to reduce tyrosine phosphorylation of the insulin receptor in insulin-stimulated hippocampal neuronal cultures [26]. Glutamate has also been shown to inhibit the IR/Akt/mTOR pathway, leading to brain insulin resistance during periods of significant mitochondrial depolarization. Mitochondrial depolarization results from excess calcium ion influx and causes insulin resistance during the IR autophosphorylation stage [27]. Therefore, the dysregulation of glutamate signaling is implicated in excitotoxicity, which is a key mechanism of neurodegeneration and brain insulin resistance [17].

Glutamate serves as the substrate for GABA, the primary inhibitory neurotransmitter in the CNS which improves protection and encourages proliferation of β-cells by activating the PI3K/Akt pathway [28,29]. It mainly affects GABergic brain regions such as the basal ganglia, thalamus, hippocampus, thalamus, and brain stem and is also secreted by β-cells in the pancreas [29]. It regulates neuronal activity by acting on the receptors GABA_A_ and GABA_B_ [30]. The GABA_A_ receptors are ionotropic and allow for rapid chloride ion influx leading to hyperpolarization. In contrast, GABA_B_ receptors G-protein coupled receptors that function as slow synaptic inhibitors [29]. It can improve encourage proliferation of β-cells by activating the PI3K/Akt pathway [28]. In addition, it has been demonstrated to attenuate insulin resistance of patients with T2D by possibly increasing IRS1 gene expression [31]. Another study demonstrated that GABA increases Glut4 expression, which also reduces insulin resistance [32]. In T2D, GABA levels and signaling are reduced in pancreatic islets, though there is counterevidence demonstrating that GABA levels in the brain are elevated in patients with T2D [33,34,35]. Alterations in GABergic signaling could tissue-specific and stage-dependent since CNS findings are relatively variable. It is possible that T2D dysregulates the excitatory/inhibitory balance between glutamate and GABA [36].

### 2.3. Role of Acetylcholine

Given its crucial role in memory and learning, acetylcholine has been extensively studied with respect to neurodegeneration and its dysregulation is implicated in AD and T2D pathogenesis (Figure 1) [18]. Nicotinic acetylcholine receptors (nACh receptors) are involved in both muscle contraction and propagating neural connections to facilitate learning and alertness. nACh receptors are widely expressed in both the peripheral and central nervous systems, where they mediate fast synaptic transmission and modulate neuronal excitability. These receptors within the central nervous system play a significant role in memory and learning, which are severely impaired in AD [37].

A recent study compared the nicotinic acetylcholine receptors in T2D mice models and controls. There were significantly lower levels of the a7 and a4 subunits in the hippocampus of the T2D models [38]. Because of the role of acetylcholine in the regulation of insulin secretion and the role of insulin in the regulation of acetylcholine transferase levels, these findings suggest that AD and T2D likely stem from or are related to the insulin resistance [39]. While this does not establish causation, it adds to the growing body of evidence linking T2D and AD.

### 2.4. Role of Mitochondria

Another common theme in both T2D and AD is mitochondrial dysfunction which suggests a broader metabolic component to neurodegeneration [3,40]. Mitochondrial dysfunction has been implicated in metabolic dysfunction, aging, and several degenerative diseases, including AD. Mitochondrial dysfunction impairs beta-oxidation, causing a buildup of lipid intermediates which disrupt the function and composition of mitochondria [11]. These fatty acid metabolites can impair insulin signaling in T2D by increasing serine phosphorylation of IRS proteins [41]. Lipid overaccumulation can activate the stress response of the mitochondria which increases ROS production. Increased oxidative stress further promotes inflammation, a key factor in both AD and T2D [11].

In the brain, mitochondria play a critical role in neuronal cell health, as neurons rely heavily on mitochondrial oxidative phosphorylation for ATP production as it is responsible for around 90% of oxygen-dependent ATP production, highlighting the importance of properly functioning mitochondria in the central nervous system and the prevention of neurodegeneration [42].

In both T2D and AD, mitochondrial dysfunction can result in increased peripheral and central insulin resistance, respectively, as well as inflammation and metabolic dysfunction [43]. Despite the differences in disease context, the overlapping mitochondrial impairments in T2D and AD underscore their interconnected pathophysiology.

### 2.5. Relationship to Insulin Signaling

Emerging evidence emphasizes the connection between insulin signaling, diabetes, and the progression of AD [3]. Recent studies have shown that elevated levels of amylin, a hormone co-secreted with insulin by the β-cells of the pancreas, can contribute to the formation of Aβ plaques in the brain [3,44]. Amylin dyshomeostasis, as evidence by amylin aggregates in the brain, was observed in patients with T2D and dementia [45]. Because amylin is involved in regulating hunger and blood sugar levels, both of which are dysregulated in T2D, the interplay between insulin and amylin release, may be a key factor linking these conditions (Figure 1) [46].

Brain insulin resistance is increasingly recognized as a contributor to AD pathophysiology. In an in vivo mouse study, brain insulin resistance was measured by examining the ratio of the insulin receptor (INSR)α-A to INSRα-B isoforms of the insulin receptor, revealing increased brain insulin resistance in AD models [47]. Another study showed that Aβ acts as a competitive inhibitor of insulin, further contributing to insulin resistance [48]. Notably, the hippocampus is a critical brain region for memory and experiences a 25% reduction in glucose metabolism in patients with AD [3]. These findings support the concept of AD as T2D and reinforce the metabolic link between T2D and AD.

**Figure 2:**
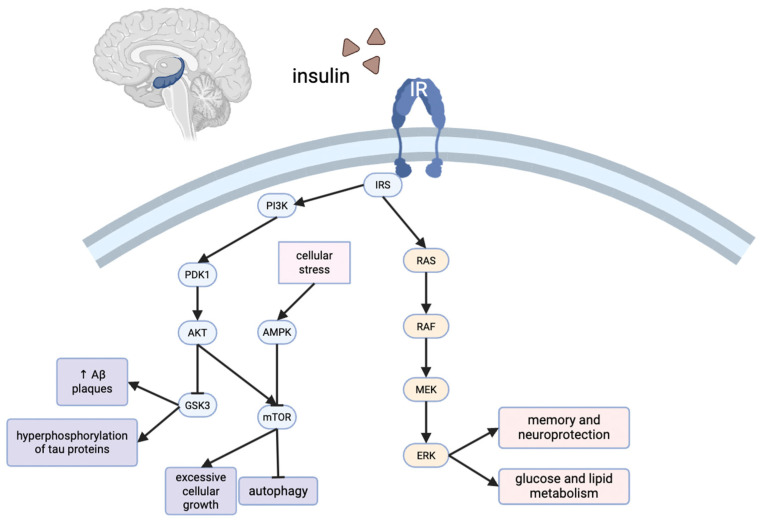
Insulin signaling in the hippocampus results in the activation of the PI3K/Akt pathway as well as the MEK/ERK pathway. Arrowheads represent activation, whereas blunted (T-shaped) lines indicate inhibition.

There are a number of cellular mechanisms associated with insulin signaling and metabolism in the brain. The activation of insulin receptors (IRs) causes the phosphorylation of IRS proteins, which function as adaptor molecules to recruit downstream effectors. These IRS proteins serve as scaffolds for key signaling pathways, including the PI3K/Akt axis, the mitogen-activated protein kinase (MAPK)/extracellular signal-regulated kinases (ERK) pathway, and the mTOR pathway (Figure 2) [49]. However, the excessive phosphorylation of these proteins on serine residues, which can be triggered by inflammatory signals, oxidative stress, or lipid accumulation, results in reduced IR binding sensitivity. This post-translational modification contributes to the disruption of insulin signaling and the development of insulin resistance [49,50].

The PI3K/Akt pathway regulates glucose transporter (Glut)3 transporters and phosphofructokinase-1 expression. Glut3, which is largely expressed in neurons, is crucial for the regulation of glucose and energy levels due to its relatively low Km. The activation of the PI3K/Akt pathway also leads to the translocation of Glut4 to the membrane of neurons in the hippocampus, ultimately enhancing cognitive performance [51].

Activation of PI3K can also activate the MAPK/ERK pathway, which is associated with memory and neuroprotection. The MAPK pathway also regulates glucose and lipid metabolism in the mediobasal hypothalamus and the dorsal vagal complex [52]. The MAPK pathway can lead to neuroinflammation that can result in accumulation of amyloid plaques, perpetuating AD symptoms [53].

The mTOR, another target of PI3K/Akt signaling, negatively regulates autophagy. The inhibition of mTOR could have neuroprotective effects in AD by promoting the removal of damaged cells or mitochondria [54]. Furthermore, the inability to perform autophagy is a common feature in neurodegenerative diseases such as AD.

The AMPK pathway is another key regulator of metabolic homeostasis. The activation of AMPK results in the inhibition of mTORC1, preventing excessive cellular growth and promoting autophagy. It leads to the suppression of gluconeogenesis, attenuating hyperglycemia. It enhances glucose uptake, facilitated by Glut4 translocation, which translates to increased glycolytic flux and ATP production. It also suppresses inflammation, which plays a key role in both T2D and AD pathophysiology [55]. In the hypothalamus, insulin and glucose inhibit AMPK activity [56]. However, AMPK is also activated by cellular stress, which can convey neuroprotective properties in some cases, but be problematic in others, depending on the severity and duration of activation. Thus, the AMPK pathway has significant effects on metabolic signaling and neuroprotection [56].

## 3. Insulin Resistance in the Brain

### 3.1. Central Insulin Resistance as the Defining Feature of T3D

Insulin resistance in the brain represents the central pathological feature of T3D [57]. Rather than emerging as a secondary complication of systemic disease, brain insulin resistance is increasingly recognized as a primary event contributing to the onset and progression of AD and related neurodegenerative conditions [58]. Regions such as the hippocampus, prefrontal cortex, hypothalamus, and olfactory bulb exhibit high densities of insulin receptors and are particularly vulnerable to disruptions in insulin signaling [59]. Consequentially, these same regions are among the earliest to exhibit metabolic dysfunction and structural degeneration in AD [60].

Neuronal insulin signaling is critical for maintaining synaptic integrity, neuroplasticity, mitochondrial homeostasis, and metabolic regulation [61]. Beyond their role in facilitating glucose uptake, as discussed in Section 2.5, activation of the PI3K/Akt and MAPK/ERK pathways also promotes long-term potentiation and protects against neuronal apoptosis through suppression of glycogen synthase kinase 3 beta (GSK-3β) [9]. Under physiological conditions, GSK-3β promotes apoptosis by phosphorylating and inactivating anti-apoptotic proteins such as Bcl-2 and Mcl-1, while simultaneously enhancing pro-apoptotic signaling through Bax and Bad activation [62]. GSK-3β also facilitates mitochondrial outer membrane permeabilization, leading to cytochrome c release, apoptosome formation, and downstream caspase-9 and caspase-3 activation [63]. In addition, GSK-3β regulates transcription factors including p53 and NF-κB, tipping the balance toward pro-apoptotic gene expression [64]. Thus, inhibition of GSK-3β preserves mitochondrial integrity and attenuates caspase-dependent neuronal apoptosis [65]. In states of insulin resistance, these signaling pathways become impaired, resulting in reduced ATP production, accumulation of ROS, mitochondrial dysfunction, and synaptic degeneration [66]. These molecular and cellular consequences of insulin resistance closely mirror the core features of early AD pathology, including energy failure, oxidative stress, and loss of neuronal connectivity [67].

### 3.2. Neuroimaging and Molecular Evidence

Functional neuroimaging studies provide compelling support for the clinical relevance of brain insulin resistance [61,68]. Fluorodeoxyglucose positron emission tomography (FDG-PET) has consistently demonstrated reduced cerebral glucose metabolism in the hippocampus, posterior cingulate cortex, and parietotemporal regions in individuals with mild cognitive impairment (MCI) and early-stage AD [69]. These metabolic changes are often detectable before the onset of measurable cognitive deficits or structural atrophy, suggesting that changes in cerebral metabolism represent early biomarkers of disease progression.

As outlined previously in Section 2.5, AD is characterized by impaired insulin signaling, including reduced expression of insulin and IGF-1 receptors and altered phosphorylation of insulin receptor substrates (IRS) that shifts from tyrosine to serine residues [70,71,72]. Within the context of neuroimaging and molecular analyses, these findings provide direct evidence that insulin resistance is not limited to peripheral tissues but manifests in the brain itself. Importantly, such receptor and IRS abnormalities have been observed in AD patients without type 2 diabetes, reinforcing the concept that central insulin resistance may arise independently and precede systemic metabolic dysfunction [73]. Cerebrospinal fluid (CSF) biomarkers further support the association between insulin resistance and AD pathology. Altered CSF concentrations of insulin, IRS signaling intermediates, and downstream effectors correlate with reduced levels of amyloid-beta 42 (Aβ42) and elevated total tau and phosphorylated tau, linking insulin resistance mechanistically to the pathogenesis of protein aggregation [74].

### 3.3. Peripheral–Central Interactions and the Pathogenic Feedback Loop

A bidirectional relationship exists between peripheral metabolic dysfunction and central insulin signaling. Chronic hyperinsulinemia, as observed in T2DM and obesity, downregulates insulin receptor expression at the blood–brain barrier (BBB), limiting insulin transport into the central nervous system and exacerbating cerebral hypoinsulinemia [74,75]. Concurrently, systemic inflammatory mediators, particularly tumor necrosis factor-alpha (TNF-α) and interleukin-6 (IL-6), can impair neuronal insulin signaling by promoting serine phosphorylation of IRS-1 and IRS-2 [76].

In parallel, elevated circulating levels of lipotoxic metabolites such as ceramides and diacylglycerols readily cross the BBB and contribute to the neuroinflammatory milieu [77]. Ceramides serve as critical bioactive sphingolipids that mechanistically bridge systemic metabolic dysfunction with central neuroinflammation. Within glial cells, activation of neutral sphingomyelinase-2 (nSMase2) catalyzes ceramide formation, leading to the assembly of ceramide-enriched membrane microdomains that facilitate Toll-like receptor 4 (TLR4) clustering and downstream signaling [78,79,80]. This structural reorganization enables recruitment of myeloid differentiation primary response 88 (MyD88) and subsequent activation of the IκB kinase (IKK) complex, promoting NF-κB nuclear translocation and transcriptional priming of pro-inflammatory genes such as TNF-α, IL-6, pro-IL-1β, and NOD-like receptor protein 3 (NLRP3) [81,82,83,84]. Concomitantly, ceramide accumulation within mitochondria disrupts electron transport and elevates reactive oxygen species (ROS), providing the secondary activation signal required for NLRP3 inflammasome assembly [85,86]. The resulting NLRP3- apoptosis-associated speck-like protein (ASC)-caspase-1 complex converts pro-IL-1β and pro-IL-18 into their mature cytokine forms, amplifying neuroinflammatory cascades in microglia and astrocytes [87,88]. Experimental suppression of ceramide synthesis or SMase activity correspondingly reduces NF-κB signaling and cytokine release, underscoring the causal role of ceramide-TLR4 coupling in immunometabolic signaling [89,90]. Furthermore, short-chain and plant-derived ceramides have been demonstrated to cross the BBB following systemic administration, detected by liquid chromatography-mass spectrometry and fluorescence tracing in rodent models [77,91]. This permeability provides a mechanistic pathway through which peripheral ceramide excess may influence central immune tone, linking metabolic lipotoxicity to neuroinflammatory progression in Alzheimer’s disease and other neurodegenerative conditions.

Mechanistically, ceramide accumulation within mitochondria disrupts electron transport and elevates reactive oxygen species, providing an oxidative signal that promotes both neuronal injury and inflammasome activation [85,92]. They also disrupt mitochondrial function and impair synaptic plasticity, thereby worsening neuronal insulin resistance [93]. Importantly, ceramide accumulation has been linked to amyloidogenic processing of APP and Tau hyperphosphorylation, both of which are hallmarks of AD pathology [94]. In particular, ceramide-mediated stabilization of beta-secretase 1 increases β-cleavage of APP, while impaired insulin/IGF-1 signaling reduces Akt-mediated inhibition of GSK-3β (glycogen synthase kinase 3β), promoting hyperphosphorylation of Tau and neuronal apoptosis [72,95]. At the molecular level, steroids interfere with insulin receptor trafficking to the neuronal membrane, inhibit PI3K/Akt signaling, and activate GSK-3β, thereby exacerbating Tau phosphorylation and neuronal apoptosis [96]. Glucocorticoid-induced GSK-3β activation and stress-hormone–mediated Tau release further potentiate neurotoxicity in hippocampal neurons [97]. Additionally crosstalk with sphingolipid metabolism sustains a self-amplifying cycle of lipid dysregulation and neuroinflammation [98]. The combined effect of systemic inflammation, BBB transport dysfunction, and direct lipid-mediated neuronal injury therefore establishes a pathogenic feedback loop that reinforces and accelerates a brain insulin resistance and contributes to Alzheimer’s disease progression [99]. Systemic cytokines and BBB transport defects have been shown to impair central insulin signaling, thereby linking peripheral inflammation with neuronal insulin resistance [100].

### 3.4. Mechanistic Contributions to Amyloid and Tau Pathology

Impaired insulin signaling plays a critical role in the accumulation of amyloid-beta and the hyperphosphorylation of tau, the two defining pathological lesions of AD [101]. The insulin-degrading enzyme (IDE), responsible for the clearance of both insulin and Aβ, becomes competitively inhibited in hyperinsulinemic states [102]. This diversion of IDE activity favors the accumulation of extracellular Aβ, contributing to plaque formation. Concurrently, loss of insulin-mediated inhibition of GSK-3β leads to increased phosphorylation of tau, resulting in neurofibrillary tangle formation and destabilization of the neuronal cytoskeleton [103].

At the cellular level, metabolic stress drives sphingolipid remodeling: ceramides accumulate via de novo synthesis (serine + palmitoyl-CoA) and sphingomyelinase-dependent hydrolysis, then reorganize the plasma membrane into ceramide-rich lipid rafts that cluster TLR4 with the adaptor proteins MyD88 and TRIF, potentiating IKK–NF-κB activation and glial cytokine release (TNF-α, IL-1β, IL-6) [104,105]. Ceramides also activate protein phosphatase 2A (PP2A) and protein kinase C ζ (PKCζ), promoting Akt dephosphorylation and disinhibiting GSK-3β, which reinforces tau hyperphosphorylation and pro-apoptotic signaling in neurons [106,107]. Parallel lipotoxic signals from diacylglycerols activate conventional/novel PKC isoforms (e.g., PKCε/θ), leading to IRS-1 serine phosphorylation, impaired PI3K/Akt transduction, and neuronal insulin resistance that further removes tonic restraint on GSK-3β [108,109]. Within microglia and astrocytes, ceramide-enriched membranes and ER stress at mitochondria-associated membranes (MAMs) increase Ca^2+^ transfer to mitochondria and mitochondrial ROS/mtDNA release, which prime and activate the NLRP3 inflammasome to mature IL-1β/IL-18 (“mito-inflammation”), sustaining a feed-forward loop of neuroinflammation [110,111]. Experimental models also show select ceramide species can traverse or accumulate across a metabolically stressed BBB, thereafter integrating into neural/glial membranes to amplify mitochondrial dysfunction and inflammasome signaling, linking peripheral lipotoxicity to central pathology [91,112].

These mechanisms position insulin resistance not only as a contributor but potentially as a driver of the molecular events that underlie neurodegeneration. The convergence of insulin signaling dysfunction with protein aggregation reinforces the hypothesis that T3D represents a metabolically driven neurodegenerative process.

### 3.5. Evidence from Preclinical Models

Experimental models have been instrumental in elucidating the consequences of isolated brain insulin resistance [113]. The intracerebroventricular streptozotocin (ICV-STZ) model, which selectively impairs insulin signaling in the brain without altering peripheral glucose homeostasis, reliably induces cognitive deficits, synaptic loss, amyloid accumulation, and tau hyperphosphorylation [114]. These phenotypes closely replicate the pathology observed in sporadic AD.

Crucially, these models demonstrate that brain insulin resistance is reversible. Intranasal administration of insulin has been shown to restore insulin signaling pathways, reduce amyloid and tau burden, improve mitochondrial function, and reverse cognitive impairment [115]. These findings suggest that central insulin resistance is not a fixed state, but one that can be therapeutically modulated.

### 3.6. Clinical Trials and Translational Opportunities

Human trials have provided promising but nuanced evidence supporting the use of intranasal insulin in patients with MCI and early AD [116]. A four-month randomized controlled trial demonstrated that daily administration of 20 IU intranasal insulin improved memory recall, preserved regional glucose metabolism as measured by FDG-PET, and favorably modified CSF Aβ42/tau ratios [117,118]. These benefits were most pronounced in apolipoprotein E ε4 (apoE ε4)-negative participants, suggesting a potential genetic basis for therapeutic responsiveness [117]. Beyond apoE ε4, other polymorphisms in genes present small risk effects for T2D and AD, though SORCS1 is the only gene to be linked to both diseases [59].

A subsequent trial comparing regular insulin to the long-acting analog detemir found greater cognitive benefit with the shorter-acting formulation [119]. Notably, higher insulin doses (60 IU) were not associated with increased efficacy and in some cases resulted in cognitive decline, indicating that optimal dosing is critical to therapeutic success [120].

Although a larger 12-month trial was discontinued due to device malfunction, its preliminary findings reaffirmed the safety of intranasal insulin delivery and highlighted its potential for disease modification) [121]. Ongoing studies are exploring combination regimens incorporating intranasal insulin with peripheral insulin sensitizers such as metformin and glucagon-like peptide-1 receptor agonists [117]. These combinatorial approaches aim to address both central and systemic contributors to cognitive decline.

Current adverse effects of intranasal insulin administration include upper respiratory infections, headaches and associated imbalance, hypoglycemia, rashes or dermal abrasions, and gastrointestinal irritation [122].

There are also limitations to the use of intranasal insulin, most notably its lack of bioavailability contributing to low permeability in the nasal mucosa and the possibility of degradation. Low permeability stems from the size of the molecules and the degradation from the prevalence of proteolytic enzymes in the nasal passage. New methods of overcoming these limitations include an adaptation of the delivery systems. Utilizing water-insoluble powders and mucoadhesive drug delivery systems can improve the longevity of intranasal insulin, and studies are ongoing in determining the best method of dispensation [122].

### 3.7. Future Directions

Insulin resistance in the brain represents a central, potentially modifiable driver of neurodegeneration [2]. It is functionally, mechanistically, and pathologically linked to the earliest changes observed in Alzheimer’s disease and other dementias [45]. The overlap between metabolic dysregulation, neuroinflammation, synaptic dysfunction, and proteinopathy supports the framing of T3D as a distinct and clinically actionable entity [123,124]. Ultimately, restoring insulin sensitivity in the brain may not only delay cognitive decline but alter the natural history of neurodegenerative disease [125].

## 4. The Cardiovascular Link

The link between cardiovascular disease, T2D, and AD has become increasingly recognized in the past decade. In fact, recent studies have highlighted a correlation between hypertension and AD [126], suggesting that vascular health may play a role in cognitive decline. This research could contribute to the development of new preventative treatments as well as diagnostic techniques aimed at reducing the risk of AD.

Insulin resistance and impaired IGF-1 signaling, both of which are characteristic of T2D and AD, are associated with atherosclerosis [126]. Diabetic dyslipidemia, a risk factor cardiovascular disease, is characterized by elevated triglycerides, reduced high-density lipoprotein cholesterol, and slightly increased low-density lipoprotein (LDL) cholesterol [127]. Dyslipidemia can exacerbate insulin resistance through the accumulation of lipid metabolites such as ceramides, which are transported in LDLs [128]. This underscores the role of cardiovascular disease risk factors such as lipid metabolism deregulation in AD pathology. Additionally, research suggests that the accumulation of Aβ can lead to diastolic dysfunction if it crosses the blood–brain barrier [126]. This aligns with the T3D hypothesis in that insulin resistance promotes amyloidogenic processes, leading to increased Aβ plaque formation, which is a key feature of both AD and cardiovascular disease [126].

The multiple connections between T2D and AD, particularly in the context of insulin resistance and cardiovascular function, suggest that it is quite plausible that AD represents a progression of T2D. The interconnectedness of body systems complicates both diagnosis and management, further highlighting the importance of understanding these connections in seeking to develop more effective strategies for early intervention and treatment.

## 5. Hormonal Connection

### 5.1. Estrogens

Numerous hormones are associated with risk and progression of AD, many of which are also closely related to the pathogenesis of T2D. These include estrogens, cortisol, and leptin [129,130]. Estrogen levels, in particular, have been correlated with dementia, cardiovascular disease, and metabolic disease risk [129,130]. The sudden decline in estrogen levels during menopause is associated with an increased risk of cognitive decline, T2D, and cardiovascular disease [129].

Estrogens play beneficial roles in insulin signaling and in pancreatic islet cell function, placing post-menopausal women with low estrogen levels at a higher risk for T2D [129]. The effects of low estrogen levels on insulin signaling do not solely affect T2D and cardiovascular disease risk but have been associated with increased cognitive decline as well [130]. Estrogen receptors in the brain, such as ER-α, which is largely found in the hippocampus, and ER-β, which is largely found in the basal forebrain and cerebral cortex, are crucial for memory [130]. Because the hippocampus is known to shrink throughout the progression of AD, leading to associated memory loss, ER-α and estrogen signaling are key areas of research.

**Figure 3:**
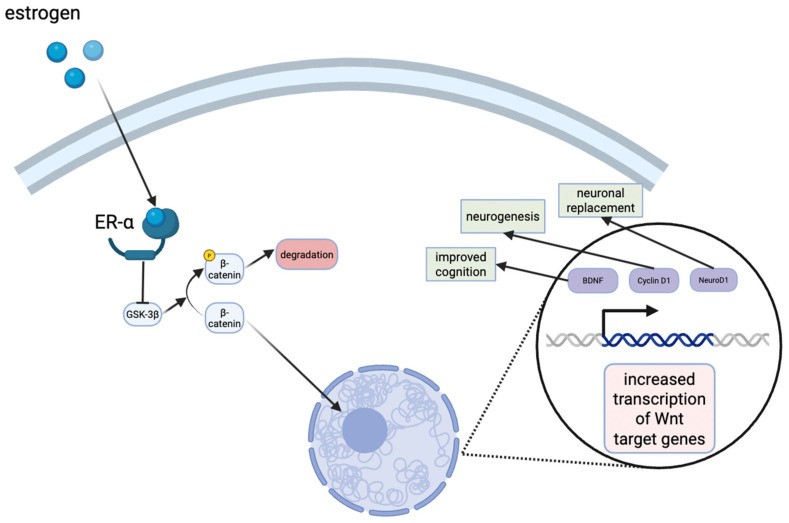
Estrogen signaling in the hippocampus results in the stabilization of β-catenin levels in the cytoplasm. The translocation to the nucleus eventually results in the transcription of Wnt target genes, such as cyclin D1, neuroD1, brain-derived neurotrophic factor (BDNF), all of which have neuroprotective effects or improve cognition.

Estrogens are known to exert neuroprotective effects, and recent studies have aimed to elucidate their mechanisms of action. For example, an in vivo rat model for vascular dementia demonstrated that estrogens protected the dorsal hippocampus CA1 region, a crucial area for learning and memory [131]. Although this model was not specific to AD, the findings are highly relevant as vascular dementia and AD share overlapping pathophysiological mechanisms, including hippocampal vulnerability, impaired cerebral perfusion, oxidative stress, and neuroinflammation [132]. Moreover, mixed dementia cases frequently exhibit both vascular and Alzheimer type pathology, underscoring the translational value of these observations [133]. In addition, estrogens have been shown to activate the Wnt/β-catenin signaling pathway in the hippocampus, which is involved in neuronal development and synaptic integrity (Figure 3) [131]. Dysregulation of this pathway has been implicated in both T2D and AD, suggesting that estrogens’ modulation of Wnt/β-catenin signaling may represent a shared protective mechanism across dementia subtypes [134].

However, high levels of estrogens, as is seen during pregnancy, can negatively affect insulin signaling and potentially lead to hyperglycemia [135]. This was demonstrated in a study showing that estradiol induced the cleavage of the INSR through a G protein-coupled estrogen receptor and the upregulation of calpain 2 expression in HepG2 cells [135]. Estradiol-induced INSR cleavage generates a soluble insulin receptor which can sequester circulating insulin and compete with membrane-bound INSRs [136]. This was reversed by metformin [135]. Collectively, these data point to the importance of regulating hormone levels, as dysregulation can lead to cognitive decline, cardiovascular disease, metabolic disease, and the subsequent progression of all three conditions.

### 5.2. Cortisol

Cortisol is a lipophilic glucocorticoid and is therefore able to cross the blood–brain barrier and bind to receptors in the brain [137]. High cortisol levels have been associated with T2D, AD, and stress [138]. The stress response is largely controlled by the endocrine, immune, and autonomic nervous systems [138]. In response to stress, corticotrophin-releasing hormone (CRH) is first released, stimulating the release of adrenocorticotropic hormone (ACTH) from the pituitary gland [138]. ACTH then binds to the ACTH receptor or type 2 melanocortin receptor, stimulating the release of cortisol [138]. The negative feedback is regulated by the glucocorticoid receptors to which cortisol binds [138].

The hippocampus expresses both types of cortisol receptors, mineralocorticoid receptors (MRs) and glucocorticoid receptors (GRs) [137]. Cortisol enhances cognitive function and memory up to the point of MR saturation, beyond which excessive GR activation leads to cognitive decline [137]. In response to stress, activation of the hypothalamic–pituitary–adrenal (HPA) axis stimulates cortisol release from the adrenal cortex [139,140]. Cortisol increases gluconeogenesis and raises blood glucose levels, while the sympathetic nervous system, activated in parallel, releases adrenaline and noradrenaline to acutely increase heart rate and energy availability [141,142]. However, chronic stress and prolonged cortisol overactivation contribute to T2D and hypertension, both of which are linked to the development of AD [143].

The correlation between cortisol and glucose levels further supports the argument for designating AD as T3D. A study of healthy 75-year-olds showed that higher fasting morning cortisol and lower folate levels were the two most accurate predictors of AD risk [144]. Another study showed higher cortisol levels in patients with AD, which correlated with smaller left hippocampal volume and lower gray matter volumes [145]. Additionally, numerous studies have demonstrated that elevated cortisol levels are associated with increased oxidative stress, Aβ plaque accumulation, and tau phosphorylation [137], all of which are hallmarks of AD pathology. Chronic stress can remodel neuronal architecture by inducing dendritic shrinkage of hippocampal CA3 and dentate gyrus neurons [146]. Glucocorticoids can also repress hippocampal neurogenesis, which is an important event in the initial progression of AD [147,148]. These findings suggest that cortisol could serve as a valuable biomarker for AD, thus highlighting the potential pros of monitoring cortisol levels as part of early interventions strategies.

### 5.3. Leptin

Leptin, which is produced by adipose tissue, plays a crucial role in regulating satiety and insulin sensitivity. However, when dysregulated, it can contribute to T2D and AD [149]. When properly regulated, leptin enhances insulin sensitivity by activating lipolysis and inhibiting lipogenesis [149]. It also activates the Janus kinase 2 (Jak2)/Signal transducer and activator of transcription 3 (STAT3) pathway, while also suppressing SOCS3 signaling [149].

In addition to its metabolic functions, leptin also has neuroprotective effects. In vitro studies using the human neuroblastoma cell line SH-SY5Y have shown that leptin can decrease the production of Aβ [149], suggesting a potential protective role in AD. However, extremely high leptin levels can lead to leptin resistance, which has been implicated in the progression of T2D and AD [150]. Leptin resistance is often found in patients with T2D in conjunction with the insulin resistance [150]. Studies have shown a correlation between elevated circulating leptin levels and increased T2D risk [150]. Hyperleptinemia is also associated with cardiovascular disease and obesity [151]. Leptin resistance leads to increased Aβ plaque formation and cognitive decline as its neuroprotective effects are negated [151].

Reducing circulating leptin levels has shown great promise in improving leptin sensitivity in hypothalamic neurons and in improving insulin sensitivity [151]. However, many patients with AD suffer from leptin deficiency, which can be detrimental due to the ability of leptin to reduce tau phosphorylation and lower Aβ levels by regulating AMPK and glycogen synthase kinase-3 [152].

Monitoring and optimizing leptin levels is essential, as both excess (indicating resistance) and deficiency can contribute to neurodegeneration. Maintaining optimal hormone levels, including estrogens, cortisol, and leptin, is crucial for the mitigation of diseases like T2D and AD, though this poses several challenges for postmenopausal women and is often not feasible without medical intervention. Additionally, the similarities in how these hormones affect both diseases and their correlation to insulin resistance further supports the concept of AD as T3D.

## 6. The Gut–Liver–Brain Connection

### Major Hallmarks of Alzheimer’s Disease

The gut microbiota–liver–brain axis is a bidirectional network which extends to neuroendocrine, metabolic, and immune pathways of communication [153]. The imbalance of gut microbiota function and composition, known as gut dysbiosis, is implicated in the development of both T2D and AD [154]. Diet plays a significant role in the progression of diabetes and its effect on cognitive health, as evidenced by studies demonstrating that high-fat diet-induced gut dysbiosis and leaky gut contribute to AD pathogenesis [154].

The gut–liver axis is linked by the permeability of the intestinal epithelium. When gut dysbiosis occurs, intestinal permeability increases which allows bacteria and their metabolites to pass into the bloodstream and enter the liver. The gut-derived components can induce or exacerbate chronic liver diseases. Endogenous alcohol production also increases as a result of gut dysbiosis, further linking the gut microbiota–liver axis [153].

Bacteria-derived metabolites such as short-chain fatty acids (SCFAs) are suspected to play a key role in the gut microbiota–brain axis and facilitate neuro-immunoendocrine regulation. Most notably, SCFAs help maintain intestinal barrier integrity, drive the maturation and function of microglia, and regulate the secretion of gut hormones [155]. Furthermore, SCFAs are transported to the central nervous system and cross the BBB where they can decrease circulating pro-inflammatory cytokines. The reduction in lipopolysaccharide-induced neurological inflammation by SCFAs has also been demonstrated in primary microglia and the hippocampus [153].Another study demonstrated that lower serum concentrations of acetate, a SCFA produced from fructose metabolism, were observed in patients with AD [156]. Additionally, decreased levels of seven SCFAs (acetic acid, butyric acid, propionic acid, formic acid, valeric acid, isolvaleric acid, and 2-methylbutyric acid) were as strong predictor of the conversion from mild cognitive impairment to AD [155]. Despite the potential benefits of SCFAs on host metabolism and neuroinflammation, the impact of these bacteria-derived metabolites is controversial. For example, acetate helps induce hepatic de novo lipogenesis which contributes to hepatic steatosis. Hepatic steatosis is characterized by excess lipid accumulation and is associated with insulin resistance [157,158]. Furthermore, SCFAs stimulate the secretion of gut–brain signaling hormones such as glucagon-like peptide-1 (GLP-1) and gastric inhibitory polypeptide (GIP) [159,160,161]. These are the primary incretin hormones secreted to stimulate insulin secretion from pancreatic β-cells [162]. Together they simulate the incretin effect which is the amplification of insulin secretion after oral glucose compared to intravenous glucose [163].

Diet plays a significant role in the progression of diabetes and its effects on cognitive health by influencing the gut microbiota–liver–brain axis. Dietary compounds are metabolized by the gut microbiota to increase their bioavailability and maximize their therapeutic potential. For instance, luteolin, a flavonoid found in many plants and herbs, has been shown to decrease brain insulin resistance in vivo by decreasing tau phosphorylation and neurofibrillary tangles. It crosses the blood–brain barrier and reduces inflammation by interacting with MAPK, NF-κB, and STAT-3 signaling pathways [164]. Additionally, luteolin has been found to activate the IGF-1 signaling cascade and induce CREB phosphorylation, which enhances insulin sensitivity in the brain. The modification of luteolin by gut microbiota is crucial for optimization of its anti-diabetic and neuroprotective effects. For example, some gut microbiota can hydrolyze luteolin glycosides into luteolin aglycones, thus altering its bioavailability and potentially its therapeutic benefits [164]. Similar processes are characteristic of other polyphenolic compounds, such as quercetin, which exhibit comparable health benefits.

## 7. Clinical and Behavioral Interventions

### 7.1. Exercise

Another crucial yet often overlooked preventative measure for AD and T2D is exercise (Figure 1). While the benefits of exercise are widely recognized, it may seem like a minor step when confronted with the formidable challenge of AD. However, numerous studies have demonstrated that exercise can significantly enhance brain health and cognitive function. An in vivo mouse study showed a reduction in Aβ levels in the hippocampus after just 3 weeks of running [164]. Another clinical study involving patients with obesity who have sedentary lifestyles demonstrated improved brain insulin action, cognitive benefits, and improved hippocampal function following an 8-week exercise program [16]. Finally, a study in mice showed that exercise promoted the transport of insulin across the blood–brain barrier and promoted its binding within the brain [165]. This is especially important because, if AD is considered a form of T3D, the ability of exercise to combat insulin resistance in the brain could be pivotal in preventing or slowing AD progression, especially in its early stages.

Exercise can significantly reduce cortisol levels, thereby slowing the accumulation of Aβ plaques and alleviating the associated oxidative stress [135]. Even small efforts to reduce stress, whether through mindfulness or exercise, can have substantial benefits by lowering cortisol levels and mitigating the effects of chronic stress [97].

### 7.2. Diet

Diet plays a critical role in overall health and can significantly impact the progression and management of T2D and, by extension, AD as T3D (Figure 1). Nutritionally induced insulin resistance has been shown to affect brain insulin resistance [166]. For instance, feeding fructose to hamsters demonstrated the neurological effects of dietary sugars [166]. An in vivo mouse study showed that a long-term high-fat diet led to brain insulin resistance and increased levels of Aβ plaques in the brain [167]. Another mouse study showed insulin resistance in the cerebral cortex of mice fed a high-fat diet [168]. The diet also led to increased serine-phosphorylated IRS-1 indicating insulin resistance, and a decreased expression of PSD-95, a protein related to post-synaptic densities [168]. Thus, a high-fat diet promotes brain insulin resistance and cognitive decline.

While the negative effects of poor diet are numerous and well-documented, a nutrient- and antioxidant-rich diet offers substantial benefits. For example, prebiotics isolated from acorn and sago have been shown to mitigate the adverse effects of a high-fat diet, attenuating insulin resistance and poor glucose metabolism [169]. An in vivo study in mice highlighted the cognitive health benefits of phenolic compounds such as thymol, isolated from medicinal herbs, which counteracted high-fat diet-induced insulin resistance, reduced Aβ plaques, and diminished tau phosphorylation [170]. There were also fewer instances of measurable cognitive impairments as was revealed by behavioral testing [170]. Thymol is thought to achieve these effects through the activation of the nuclear factor erythroid 2-related factor 2/Heme oxygenase-1 (Nrf2/HO-1) pathway [170].

The idea that the consumption of certain foods can be linked to anti-diabetic effects has led to the discovery and development of therapeutic applications and delivery systems of plant-derived bioactive compounds. Exosome-like nanoparticles have recently shown promise for health benefits. A study isolated these from garlic and tested their effects on cognition, finding that they could reduce high-fat diet-induced brain inflammation as was demonstrated by diminished levels of pro-inflammatory cytokines such as IFN-γ and TNF-α [171]. Additionally, improved glucose tolerance, insulin sensitivity, and memory function were observed [171]. Another in vivo mouse study showed that *Nigella sativa* oil could improve insulin resistance and prevent both amyloid plaque formation and tau phosphorylation, both of which are hallmarks of AD [172]. The therapeutic overlap between T2D and AD supports the concept of T3D and suggests that anti-diabetic agents could hold promise as potential treatments for AD.

Polyphenols have been shown to offer numerous health benefits, especially related to reducing inflammation and oxidative stress in T2D [173]. An in vivo rat study showed that treatment with *Boswellia serrata* gum inhibited Aβ plaque formation and tau phosphorylation, increased insulin sensitivity, lowered cytokine levels, and improved oxidative stress in the hippocampus [174]. Polyphenols from *Boswellia serrata* gum are likely responsible for these neuroprotective effects [174].

Overall, the reviewed literature shows that dietary modifications, including the introduction of certain phenols or polyphenols, as well as using anti-diabetic drugs or nanoparticles, could significantly slow AD progression and promote cognitive health.

### 7.3. Sleep

The health benefits of sleep are widely recognized in the field of medicine, with sleep being critical for optimal cognitive function and overall brain health [175,176]. A recent study analyzing the risk of dementia in T2D patients showed that individuals with sleep disorders had a higher risk of developing both dementia and AD [177]. This could be the result of less opportunity for the glymphatic system to remove waste from the CNS, as this system typically functions while sleeping. Another major factor related to AD is the regulation of cerebrospinal fluid levels of Aβ. These levels are higher during wakefulness and decrease during sleep. Difficulty regulating these levels could contribute to the buildup of plaques characteristic of AD. Lack of sleep can also reduce brain volume, which is another factor associated with AD [178]. Sleep deprivation is also associated with poor insulin signaling and the dysregulation of hormone and neurotransmitter levels, such as cortisol, serotonin, glutamate, and acetylcholine [179].

The impact of sleep on insulin resistance holds promise for the treatment of both T2D and AD (Figure 1). In vivo studies show an increase in PI3K/Akt/mTOR signaling in mice suffering from sleep-deprivation, resulting in an increase in Aβ levels [179]. The importance of slow-wave sleep has also been highlighted in a study that indicated lower insulin sensitivity in patients who did not experience sufficient slow-wave sleep, regardless of the overall sleep duration [180]. Obesity in T2D patients has been associated with reduced slow-wave sleep and an increased risk of sleep-disordered breathing. In turn, this sleep-disordered breathing is associated with AD. Sleep-disordered breathing, such as obstructive sleep apnea, central sleep apnea, or complex sleep apnea, can lead to decreased brain mass largely in the hippocampus, resulting in memory loss and the neurodegeneration characteristic of AD [178]. This progression from T2D to sleep-disordered breathing to neurodegeneration and memory loss further raises the question of whether AD could result from the progression of T2D.

## 8. Future Research

### 8.1. Cellular Mechanisms

Because T3D is a newly emerging idea and is currently in the early stages of research, there is much that is unknown. Because of the largely understudied nature of T3D, there are many unknown aspects of the connection that warrant future research. These directions include the connection between T2D, AD, and the potential overlap as T3D. While there are significant differences between AD and T2D, there is hesitation to fully embrace the idea of T3D as a comprehensive explanation for AD. It is possible that T3D could represent one aspect of AD, rather than encompassing all of its hallmarks. Therefore, future research should focus on understanding the specific cellular mechanisms linking insulin resistance to the progression of neurodegeneration. This could be achieved by analyzing the hallmarks of AD, such as Aβ plaque accumulation and tau phosphorylation, within the context of insulin signaling.

One of the major arguments against the concept of T3D centers around whether insulin resistance directly causes the hallmarks of AD, or if the dysregulation resulting from AD leads to insulin resistance as a secondary effect. This remains a significant gap in the literature. Because of this, future research should be directed towards determining the sequence of events between the development of AD and its hallmarks, and how insulin resistance in the brain may fit into this pathway.

### 8.2. Medical Interventions

If insulin resistance in the brain is indeed a key contributor to AD, future research could be directed towards the development of targeted treatments or preventative strategies for AD. This could be accomplished by targeting insulin receptors or identifying compounds capable of crossing the blood–brain barrier and mimicking the effects of insulin. This could eventually lead to personalized treatments for AD, tailored to individuals who are most affected by insulin resistance. Finally, research could explore methods for early screening and prevention of AD by measuring insulin resistance in the brain. The long-term goal would be to conduct clinical trials that assess the effects of insulin resistance-targeting interventions in AD patients, or those who are at a high risk of developing AD.

A number of similarities exist between AD and T2D, and the list continues to grow (Figure 1). These similarities, when considered in isolation, could be written off as coincidental or as the hallmarks of multiple diseases. However, this argument has been used to challenge the concept of T3D. It is a valid point, as many of these hallmarks, including mitochondrial dysfunction and inflammation, are seen in numerous diseases and health conditions. However, the critical question remains: does insulin resistance in the brain have the capacity to directly cause the major hallmarks of AD, including neurofibrillary tangles, tau hyperphosphorylation, and Aβ plaques? If this is indeed the case, it would significantly shift our understanding of AD, as well as open new avenues for medical advancements and potential interventions. Therefore, future research should aim to determine the causative effects of brain insulin resistance and whether these are compelling enough to justify labeling AD as T3D.

## 9. Concluding Remarks

The pathogenesis of T2D and AD shares several similarities, including systemic insulin resistance (classically involving skeletal muscle, adipose tissue, and liver) as well as resistance in the pancreas and brain, along with metabolic dysregulation and hormonal imbalances. AD is characterized by the accumulation of Aβ plaques, increased tau phosphorylation, and decreased hippocampal volume, all of which contribute to cognitive decline and memory loss. Studies have shown that elevated levels of amylin, a hormone co-secreted with insulin by the pancreas, may contribute to the formation of Aβ plaques [3,44]. Additionally, insulin and IGF-1 resistance in the hippocampus have been linked to the loss of hippocampal mass in AD models [3,181].

Hormone regulation plays a critical role in the progression of both T2D and AD. Declining estrogen levels, particularly during menopause, can contribute to cognitive decline and insulin resistance due to the role of estrogens in insulin signaling [3,129,181]. Estrogen receptors are highly concentrated in the hippocampus, the major site of degeneration in patients with AD [3,129,130,181]. Cortisol also influences insulin signaling and the regulation of blood glucose levels. Elevated cortisol levels are associated with an increased risk of both AD and T2D, as well as heightened Aβ plaque formation, oxidative stress, and tau phosphorylation [137]. Additionally, dysregulation of leptin is commonly observed in patients with T2D. This has also been implicated in AD. Leptin resistance or low leptin levels diminish its neuroprotective effects, leaving the brain more vulnerable to neurodegeneration [151]. Monitoring and regulating these hormone levels could be a useful strategy for the diagnosis and prevention of both AD and T2D, further demonstrating the interconnected nature of T2D and AD and their potential therapeutic overlap.

Behavioral interventions, including exercise, diet, and sleep, have been demonstrated to be effective in preventing or slowing AD progression. Exercise has been shown to decrease cortisol levels, improve hippocampal function, and enhance overall brain health [16]. There is also strong evidence that exercise can attenuate Aβ plaque buildup and improve brain insulin sensitivity [182].

Diet also plays a critical role in maintaining cognitive health, as high-sugar or high-fat diets have been linked to increased brain insulin resistance and neurodegeneration [168]. However, the addition of phenols, nanoparticles, or even certain anti-diabetic dietary supplements has been shown to mitigate insulin resistance, reduce inflammation and tau phosphorylation, diminish Aβ plaque accumulation, and enhance cognitive function [169,172].

Sleep is also a crucial factor, as sleep deprivation has been shown to increase Aβ levels, increase cortisol levels, decrease brain mass, and heighten insulin resistance. Furthermore, T2D can increase the risk of sleep-disordered breathing, which has been linked to cognitive decline and neurodegeneration that is characteristic of AD. Early adoption of healthy lifestyle choices, including exercise, balanced nutrition, and adequate sleep, especially early in life, can provide long-term benefits, potentially preventing neurodegeneration and promoting cognitive health well into old age.

The overlap between T2D and AD supports the use of “type 3 diabetes” as a conceptual framework, thus highlighting the role of impaired insulin signaling in the brain. However, this classification remains under active debate. Insulin resistance may represent an initiating factor, a secondary consequence, or a combined condition that worsens the progression of the disease. Future work should aim to clarify these relationships while exploring therapeutic strategies that target insulin signaling.

## Abbreviations

The following abbreviations are used in this manuscript:

T3D	Type 3 diabetes
T2D	Type 2 diabetes
AD	Alzheimer’s disease
Aβ	beta amyloid Aβ
GABA	γ-aminobutyric acid
NMDA	N-methyl-D-aspartate
AMPA	α-amino-5-methyl-3-hydroxy-4-isoxazole propionic acid
Akt	protein kinase B
mTOR	mammalian target of rapamycin
IL-	Interleukin
TNF-α	Tumor necrosis factor alpha
nSMase2	Neutral sphingomyelinase-2
TLR-4	Toll-like receptor 4
Myd88	Myeloid differentiation primary response 88
IKK	(IκB) kinase
NLRP3	NOD-like receptor protein 3
ASC	Apoptosis-associated speck-like protein
GSK-3	Glycogen synthase kinase 3
IDE	Insulin-degrading enzyme
PP2	Protein phosphatase 2
PKC	Protein kinase C
MAMs	Mitochondria-associated membranes
AMPK	AMP-activated protein kinase
INSR	Insulin receptor
PI3K	Phosphoinositide 3 kinase
ERK	Extracellular signal-regulated kinases
MAPK	Mitogen-activated protein kinase
mTORC1	Mechanistic target of rapamycin complex
IGF	Insulin-like growth factor
LDL	Low-density lipoprotein
ACTH	adrenocorticotropic hormone
MR	Mineralocorticoid receptor
GR	Glucocorticoid receptors
JAK	Janus kinase
STAT3	Signal transducer and activator of transcription
SOC3	Suppressor of cytokine signaling
GLP-1	Glucagon-like peptide-1
GIP	Gastric inhibitory polypeptide
SCFAs	Short-chain fatty acids
NF-κB	Nuclear factor kappa B
Nrf2/HO-1	Nuclear factor erythroid 2-related factor 2/Heme oxygenase-1
CREB	cAMP response element binding protein
PSD	Postsynaptic density protein
ROS	Reactive oxygen species
